# Interagency technical consultation on improving mortality reporting in Sierra Leone: meeting report

**DOI:** 10.11604/pamj.2017.26.27.11111

**Published:** 2017-01-18

**Authors:** Yonas Asfaw, Isaac Boateng, Mauricio Calderon, Grazia Caleo, Allan Conteh, Salifu Conteh, Foday Dafae, Achintya Dey, Nadia Duffy, Daffney Davies, Patrick Fatoma, John Fleming, Boima Gogra, Yelena Gorina, Anna Grigoryan, Sara Hersey, Sam Hoare, Sonnia-Magba Bu-Buakei Jabbi, Amara Jambai, Joseph Jasperse, Reinhard Kaiser, Gandi Kallon, Ansumana Kamara, Fatmata Zara Kamara, Isata Pamela Kamara, Wogba Kamara, Joseph Kandeh, Mustapha Kanu, Mabinty Kargbo, Samuel Kargbo, Richard Konie, Simeon Kuyembeh, Patrick Lansana, Fiona Mclysaght, Sheena McCann, Alhaji Samuka Nallo, Stephanie Ngai, Erin Nichols, Charles Njuguna, Shikanga O-Tipo, Sulaiman Parker, Nuzhat Rafique, John Redd, Thomas Samba, Kerry Souza, Alex Tran, Chief Mathew Gibao Younge

**Affiliations:** 1 World Health Organization, Geneva, Switzerland; 2 Manson Unit, Médecins Sans Frontières, London, UK; 3 National Social Security Insurance Trust, Sierra Leone; 4 Sierra Leone Police, Sierra Leone; 5 Sierra Leone Ministry of Health and Sanitation, Sierra Leone; 6 Centers for Disease Control and Prevention, Hyattsville, United States; 7 Virtues Funeral Homes, Sierra Leone; 8 National Ebola Response Center, Sierra Leone; 9 International Federation of Red Cross and Red Crescent Societies, Geneva, Switzerland; 10 Western Area Ebola Response Center, Sierra Leone Army, Sierra Leone; 11 Médecins Sans Frontières, Operational Centre Amsterdam, UK; 12 Statistics Sierra Leone, Sierra Leone; 13 International Rescue Committee, New York, United States; 14 Concern Worldwide, UK; 15 New York City Department of Health and Mental Hygiene, United States; 16 Freetown City Council, Sierra Leone; 17 United Nations Children’s Fund, New York, United States; 18 GOAL, Malawi; 19 Western Area Tribal Heads, Sierra Leone

**Keywords:** Sierra Leone, mortality, death, EVD, Ebola, CRVS

## Abstract

By the end of the Ebola epidemic, death reporting in Sierra Leone (SL) became more acceptable amongst local populations, with nearly all deaths being reported to the Ebola hot line alert centers. To continue the positive momentum generated by the epidemic, the Sierra Leone Ministry of Health and Sanitation (MoHS) and the US Centers for Disease Control and Prevention (CDC) organized and conducted the two-day Inter-agency Consultations on Improving Mortality Reporting in Sierra Leone (Consultations). In conjunction with the Consultations, participants were also offered a one-day, in-person training on the major components, characteristics, and uses of a national Civil Registration and Vital Statistics (CRVS) system. To understand processes used by governmental and non-governmental organizations in collection of death data before and during the Ebola epidemic, and to develop recommendations on improving death reporting and CRVS in Sierra Leone. The Inter-agency Consultations were conducted in person over two days in October, 2015. Real-time notes were kept by CDC staff for later abstraction and summarizing. Presenters agreed to share their materials (usually PowerPoint presentations) and approved the summaries. Challenges to implementation and suggestions for improving death reporting were drawn from the presentations and from anonymous suggestions collected at the end of each of three days of the Consultations. The Consultations attracted more than 80 participants from 28 Sierra Leone governmental, business, and other non-governmental organizations. Over the course of 18 presentations, participants presented and discussed the ways deaths were reported before and during the Ebola epidemic and ways in which the CRVS in Sierra Leone might be improved. The presentations made clear the need to improve death reporting in order to improve the health status of Sierra Leone. Many presenters and participants discussed the challenges to improvements, including lack of infrastructure and country diversity. In addition, participants generally agreed upon the need for improving the government’s understanding of the benefits of death reporting at multiple levels: from local chiefdom authorities and councils to the community and individual families. Despite the many challenges identified, all participants stressed the need for modernizing and improving death registration in Sierra Leone. The recommendations from the presentations and notes collected at the end of each day can be categorized within the following five domains: capacity building (organizational, staffing, infrastructure, policies, guidelines and tools), awareness and sensitization (including strategies to use best practices and emerging technologies), political will (governmental support and prioritization), funding (providing resources to achieve sustainability), and monitoring and evaluation (developing charts of existing death reporting pathways and identifying challenges).

## Meeting Report

By the end of Ebola epidemic, death reporting in Sierra Leone had become more acceptable amongst local populations, with nearly all deaths reported to the hot line alert centers. To continue the positive momentum generated by the epidemic, the Sierra Leone Ministry of Health and Sanitation (MoHS) and the U.S. Centers for Disease Control and Prevention (CDC) organized and conducted Interagency Consultations on Improving Mortality Reporting in Sierra Leone (Consultations). As a part of the Consultations, participants were offered one-day training on the major components, characteristics, and uses of a national Civil Registration and Vital Statistics. The objective of the Consultations was to understand processes used by governmental and non-governmental organizations in death data collection before and during the Ebola epidemic and to develop recommendations on improving death reporting and civil registration.

## Death reporting and registration in Sierra Leone, prior to the Ebola epidemic

During the Interagency Technical Consultations, representatives of the Sierra Leone Office of Births and Deaths (OBD), Directorate of the Primary Health Care (MoHS/PHC) presented information on the current processes for reporting and registering deaths in Sierra Leone. Existing requirements for registration of both births and deaths are established by the Births and Deaths Registration Act No. 11, enacted in 1983. Three categories of death registration with the Office of Birth and Death were described: 1. Original registration (death occurring and registered within 14 days); 2. “Late” death registration (death occurring and registered after 14 days but within one year); and 3. “Delayed” death registration (death occurring and registered after one year or more). A separate paper form exists for each type of death registration. All documents are stored within the Office of Birth and Death in the paper format. The prescribed death registration process is comprised of the following steps: a Medical Certificate of Cause of Death is prepared by a medical practitioner; the medical practitioner may or may not have attended the death. A family member or other party may report a death to a medical practitioner who then provides a medical report of death. Two types of death – certified and uncertified deaths – are described. Certified deaths are those deaths that are attended by a medical care provider, while uncertified deaths are not; a family member or other party takes the medical certificate to the Office of Births and Deaths, which prepares a Statistical Notification Form; Using information provided on the Medical Certificate, the Office of Births and Deaths prepares a Death Record and reads the record to the family member to confirm that he/she agrees with the transcribed information. The family member then signs the Death Record; the Office of Births and Deaths provides the family member with a Burial Permit. The family member can take the Burial Permit to the City/Rural/Local Council, whereby the family member will be allowed to choose a cemetery and be issued a burial plot; the Office of Births and Deaths issues a Death Certificate.

## Violent deaths

The reporting and registration of violent deaths often represents a departure from the standard, established process due to the involvement of the Police. A representative from the Sierra Leone Police Force provided information on their role in death reporting, noting that the Police have a cooperative relationship with the Office of Births and Deaths of MoHS/PHC. While only a small fraction of deaths from natural causes are reported, the Police representative noted that violent deaths are much more likely to be registered with Office of Births and Deaths following reporting to the Police. In the case of potential homicide and suicide deaths, the corpse will be transferred from the crime scene to the mortuary autopsy, thereby delaying the generation of the medical certificate of cause of death.

## Weaknesses and challenges associated with the current death registration process

Although death registration is compulsory, compliance with the regulation is very low. The Office of Births and Deaths noted the following among challenges to the reporting and registration of deaths in Sierra Leone: legal mechanisms to enforce compulsory death registration are lacking; traditional beliefs and practices surrounding death and burial contribute to a lack of death registration; outside of deaths that occur in medical settings, cause of death is difficult to ascertain; in Sierra Leone, there is low awareness of the importance of death registration; cemeteries and burial sites are not well controlled; more personnel are needed to staff death registration; the existing registration process is antiquated, hindering data collection, analysis, reporting and dissemination of death data; death data are underused by authorities.

## Recommendations for strengthening the current death registration process

The Office of Births and Deaths stressed that a work is currently in place for reporting and registering deaths and advocated for the strengthening the current process. To that end, the Office of Births and Deaths offered the following recommendations: improve civic education and community sensitization regarding the importance and uses of death registration; modernize the death registration process (to include computerized records etc.); improve and increase the capacity of registration staff; produce and enforce national policies and by-laws on compulsory death registration; improve collaboration and harmonize the registration process; review and update Births and Death Registration Act; provide adequately equipped offices, facilities, and equipment for proper records management and operations.

Representatives of the Police Force offered the following suggestions for improving registration of deaths: the majority of natural deaths are not reported in remote villages in Sierra Leone; there is need for sensitization of elders and all members of these remote villages on the importance of death registration. Traditional leaders should be involved; the Civil Registration Department of Sierra Leone should have focal persons responsible for the registration of all deaths that occur within their localities; communities should be provided trainings or workshops on how to collect information on deaths that occur in their communities; legislation should be prepared on burial processes in the country; ambulances should be provided to all Police Regional Headquarters to attend to accident or homicide cases; burial teams should be maintained, and they should expand their operational areas to chiefdom level.

## Burial practices and records

Regulations provide for a system of burial practices in Sierra Leone. Together, the Local Government Act of 2004, which established City Councils and outlined their general powers, and the Public Health Ordinance of 1960 are invoked in assigning the responsibility of City Councils to oversee burial practices. A representative of the Freetown City Council described the policy and practices associated with burials in Freetown. This process resumes where the above-described process of death registration leaves off – presenting a burial permit to the City Council. The family member must then pay a fee, depending upon the number of burial spaces and/or a vault. The Cemetery Clerk at the city council then issues a second type of burial permit – a cemetery burial permit – to the family member, authorizing interment. The family member carries this permit to the cemetery of their choice. After the burial is conducted, the burial register is signed by the Cemetery Clerk, and the grave is registered using the “Registration of Ownership of Grave Space” (or grave coding) form. A burial register compiles information from various cemeteries together. Like personnel from the Office of Births and Deaths, personnel from the Freetown City Council advocated for a continuation of burial practices that pre-dated the Ebola epidemic. The representative described the processes in place as “transparent and acceptable.” He urged partners to support the Freetown City Council in cemetery management and capacity building.

## Death reporting and registration in Sierra Leone during the Ebola epidemic

### Community death alerts: 117, Community Event-Based Surveillance (CEBS), and local alert lines

Through the end of the Ebola Virus Disease (EVD) outbreak, all community deaths in Sierra Leone were required to be reported to relevant authorities and have a Safe and Dignified Burial (SDB) due to the risk of EVD transmission. Several mechanisms were developed to support the death alert process, depending on the setting of the death and the structures in each district. In Western area, which was not covered by CEBS, community deaths were alerted by calling the 117 call center directly. Family or household members, neighbors, and/or community members or community health workers (CHW) assign to the area were generally the individuals responsible for calling in a death to 117. The 117 call center then reported the death alert to the District Emergency Response Center (DERC) for further investigation and action. In other districts, family, community members or District Surveillance Officers (DSO) reported deaths directly to 117, the local alert line, or the relevant DERC. Under the CEBS structure (in districts other than Western), community health monitors (CHMs) identified and reported deaths in their communities to the Chiefdom Surveillance Supervisor (CSS). The CSS communicated with a Community Health Officer (CHO) to screen the alert and report it to the DERC, via the local alert line or direct DERC line. CHMs also had six other EVD-related triggers for reporting, but since the inception of CEBS, the majority of reports were of any community deaths.

### Death investigations: burial DSOs, swabbing, and safe and dignified burials

Once DERC was informed of a death alert, an investigation and the SDB process was initiated. Burial and swab teams were dispatched to collect, prepare, and swab the body. The swab was sent to the laboratory for EVD testing, the results of which were reported back to the DERC and then to the family. A burial DSO was also dispatched to complete an investigation and fill out a case investigation form to determine if the death met the criteria for a suspected EVD case. The deceased was then brought to the cemetery, where the family could join for a safe funeral and observation of the burial. In Freetown, the designated cemeteries for SDB were King Tom and Waterloo cemeteries. The deceased was buried by the SDB team and information on grave number, date, name, address, age, and sex were collected and entered into the burial register at the cemetery. Most of thhese data were later entered into a spreadsheet and stored electronically. The Freetown City Council also received a report of the lab result from the swabs, certified by the Western Area Emergency Response Center (WAERC).

### Lessons learned: data collection

During the EVD outbreak, access to available mortality data informed clinical practice as well as interventions targeted toward geographic areas. Médecins Sans Frontières (Doctors Without Borders, MSF), Operational Centre Amsterdam (MSF-OCA) operated three Ebola Management Centers (EMCs) in Sierra Leone and implemented a tablet-based data collection system toward the end of the outbreak to gather information on patient admissions, clinical courses, and outcomes. MSF-OCA analyzed outcome data on confirmed EVD cases who were alive upon admission to MSF EMCs to determine risk factors for mortality. Patient age, Ct-value on admission (Ct-value is a reverse transcriptase polymerase chain reaction- based threshold cycle value to identify Ebola infection), and presence of wet symptoms all influenced risk of mortality. Pregnant women were found to be at higher risk of death, and MSF developed clinical management guidelines to support pregnant women. GOAL analyzed mortality data from 117 and local alerting systems in Port Loko and Kambia. Recognizing that the chiefdom is too large of a geographic area to target for interventions and health promotion activities, geographic sections were used to assess the expected number of deaths (based on projections made using 2004 census data) and the actual number of alerts. Based on the ratio of actual death alerts to expect number of deaths, “silent” sections and clusters of sections with apparent underreporting of mortality were identified and targeted so that responders could understand reasons for underreporting and increase outreach to improve reporting. Additionally, certain sections also reported higher than the expected number of deaths, triggering additional investigations, as this could reflect poor estimates of expected deaths and/or truly elevated mortality rates. National Social Security and Insurance Trust (NASSIT) representatives noted that the flow of applications for death benefits during the epidemic did not rise dramatically. A likely explanation for this is that the deceased were not NASSIT members. However, since the National Ebola Response Center (NERC) started issuing death certificates, the number of claims to NASSIT did somewhat increase.

## Moving forward

### Reporting

The EVD epidemic led to the development of some of the most thorough mortality reporting ever observed in Sierra Leone. Consultations presenters emphasized the value of the systems that were developed during the response and the importance of maintaining these processes and requirements after the EVD epidemic is over, particularly the requirement that all community deaths be reported. Maintenance of 117 and local alert lines will allow communities to continue to engage in familiar structures to report deaths. Additionally, as a part of the Integrated Disease Surveillance and Response (IDSR), CEBS, with adaptations, can continue to be a mechanism for mortality reporting. Community health workers are the most likely candidates to assume the responsibility of CEBS-based mortality reporting, but they are also at risk being overburdened and should be adequately trained and incentivized. With the decommissioning of the NERC and the DERCs, mortality reporting is expected to shift to Peripheral Health Units (PHUs) and MoHS’ District Health Management Teams (DHMTs) with oversight from MoHS. Additional support for data management and rapid response capacity will be needed. Under IDSR, there are also plans to transition to electronic reporting platforms. The importance of the death registration for its beneficiaries was described in the NASSIT presentations. A death certificate is required for the family member to initiate the processing of survivor benefits and to convert the benefits to a survivor’s pension. The NASSIT presentation emphasized the need for automation and decentralization of the civil registration process, training, and creation of a centralized database. In their presentation, Statistics Sierra Leone representatives called for improving the civil registration process, particularly through sensitization and training of the central, local chiefdom authorities and councils on the Civil Registration and Vital Statistics and creating a central national data warehouse with relational databases of birth and deaths. That would greatly enhance mortality reporting and reduce reliance on survey and censuses.

### Burials

Proposed surveillance policies at the time of the Consultations indicated that SDBs would only continue for deaths with a high index of suspicion for EVD after 0+42 days. All deaths will continue to be reported and investigated if EVD is suspected. Cemeteries in the Western Area maintain burial registers, which were supported by International Federation of Red Cross and Red Crescent Societies (IFRC) and Concern Worldwide during the EVD epidemic. Data on age, gender, appropriate date, and address are entered by hand and later recorded electronically on all burials conducted by IFRC and Concern Worldwide teams in Western Area. Maintenance of these systems is important to ensure ongoing data collection; burial registers could also be transitioned to electronic platforms in the future. It was suggested that the burial data systems should also be expanded to other cemeteries in the country if they do not currently exist.

### Data uses

In the absence of a formal mortality registration system, mandatory notification of all deaths provides an opportunity for data collection not only on the fact of death, but also on demographic information and sometimes cause of death. Community health workers and others at the PHU or DHMT level can be trained on verbal autopsy methods in order to obtain information from the family or community on possible causes of death. Any additional information of cause-specific mortality is valuable in assessing public health priorities and interventions in the country. Additional information on under-five mortality in Sierra Leone would also be valuable in targeting interventions to improve health outcomes among children. At the health care facility level, mortality reporting could be systematized and integrated into the Health Management Information System (HMIS). Health care facilities should have the capacity to capture information on age, sex, and cause of death, and should also support CHW programs in reporting deaths. Both community and health care facility-based reporting are important for further understanding causes of under-five and maternal mortality and for appropriately targeting interventions based on cause and geography.

## Recommendations for improving death reporting and registration

After each of day of the Consultations, the participants were asked to provide suggestions and recommendations on how to improve death registration process in Sierra Leone. Most of the participants’ suggestions and recommendations fell under the following categories: (1) Capacity building, (2) Public awareness, (3) Political will, (4) Financial resources/funding, and (5) Monitoring & evaluation.

### Capacity building

The majority of the attendees commented on the need for strengthening the Civil Registry organizational structure and staff capabilities, which might include required policies, guidelines and tools: need to build registration tools including electronic death reporting and database for death records; adequate office space and equipment - computers/laptops are needed; data quality needs to be improved; coordination with other organizations collecting similar information to avoid duplication; technical assistance, training and support from CDC and donor organizations.

Training needs were expressed for: training for Civil Registration staff in data collection, processing and analyzing; training of military and police forces to collect death data; training for Chief/chiefdom authorities on death data collection; training for medical students in mortality reporting; training of CHWs. Transportation means for the national and district level offices; increase the number of staff members for all levels (chiefdom, district, national); all cemeteries and funeral homes need to be part of the Sierra Leone death recording system; civil registration basics should be integrated into the school curriculum.

### Public awareness/Sensitization

Various strategies can be used to implement civil registration and death reporting public awareness campaign and sensitization, using best practices and emerging technologies at different levels. Benefits of timely and better-quality death reporting; Publicity/Public sensitization: using different advocacy tools and media channels to emphasize the importance of death registration (via TV, radio), distribution of printed materials such as hand bills/handouts, leaflets, etc. at the community, district, and national levels.

### Political will

Government support and prioritization in developing a functional and sustainable national birth and death reporting system compatible with international standards: legislative framework to support the existing infrastructure and logistical aspects of the death registration; by laws to enforce compulsory death registration that is free of charge; identify and emphasize the role of traditional healers in community death reporting.

### Financial Resources/Funding

Continuous financial support to develop and sustain the CRVS system in Sierra Leone is needed.

### Monitoring & Evaluation

Develop a comprehensive chart to describe the death reporting data flow; evaluate current community death reporting process and practices and identify key challenges.

### Additional material

- Pre-Ebola death recording and reporting in Sierra Leone [Under processing]
- Intermittent and survey death information collected by Statistics Sierra Leone [Under processing]
- Death Data collected during Ebola epidemic [Under processing]
- Suggestion Cards submitted during the Interagency Consultations on Improving Mortality Reporting in Sierra Leone [Under processing]
- Notes from the Interagency Consultations on Improving Mortality Reporting in Sierra Leone [Under processing]

